# Transplantation and survival of mouse inner ear progenitor/stem cells in the organ of Corti after cochleostomy of hearing-impaired guinea pigs: preliminary results

**DOI:** 10.1590/1414-431X20155064

**Published:** 2016-03-18

**Authors:** L.C.M. Barboza, K. Lezirovitz, D.B. Zanatta, B.E. Strauss, R.C. Mingroni-Netto, J. Oiticica, L.A. Haddad, R.F. Bento

**Affiliations:** 1 Departamento de Otorrinolaringologia (LIM32), Hospital das Clínicas, Faculdade de Medicina, Universidade de São Paulo, São Paulo, SP, Brasil; 2 Setor de Vetores Virais, Laboratório de Genética e Cardiologia Molecular, Instituto do Coração, Faculdade de Medicina, Universidade de São Paulo, São Paulo, SP, Brasil; 3 Laboratório de Vetores Virais, Centro de Investigação Translacional em Oncologia, Instituto do Câncer do Estado de São Paulo, Faculdade de Medicina, Universidade de São Paulo, São Paulo, SP, Brasil; 4 Departamento de Genética e Biologia Evolutiva, Instituto de Biociências, Universidade de São Paulo, São Paulo, SP, Brasil

**Keywords:** Cochlea, Hearing loss, Stem cells, Cell transplantation, Ototoxicity

## Abstract

In mammals, damage to sensory receptor cells (hair cells) of the inner ear results in permanent sensorineural hearing loss. Here, we investigated whether postnatal mouse inner ear progenitor/stem cells (mIESCs) are viable after transplantation into the basal turns of neomycin-injured guinea pig cochleas. We also examined the effects of mIESC transplantation on auditory functions. Eight adult female *Cavia porcellus* guinea pigs (250-350g) were deafened by intratympanic neomycin delivery. After 7 days, the animals were randomly divided in two groups. The study group (n=4) received transplantation of *LacZ*-positive mIESCs in culture medium into the scala tympani. The control group (n=4) received culture medium only. At 2 weeks after transplantation, functional analyses were performed by auditory brainstem response measurement, and the animals were sacrificed. The presence of mIESCs was evaluated by immunohistochemistry of sections of the cochlea from the study group. Non-parametric tests were used for statistical analysis of the data. Intratympanic neomycin delivery damaged hair cells and increased auditory thresholds prior to cell transplantation. There were no significant differences between auditory brainstem thresholds before and after transplantation in individual guinea pigs. Some mIESCs were observed in all scalae of the basal turns of the injured cochleas, and a proportion of these cells expressed the hair cell marker myosin VIIa. Some transplanted mIESCs engrafted in the cochlear basilar membrane. Our study demonstrates that transplanted cells survived and engrafted in the organ of Corti after cochleostomy.

## Introduction

Corwin and Cotanche (1) first described structural and functional regeneration of the auditory sensory epithelium in birds, fish, and reptiles after acoustic trauma or ototoxic injury. In contrast, mammalian inner ear cells are terminally differentiated during the embryonic period. Therefore, they do not re-enter the cell cycle, and sensorineural hearing loss is irreversible.

Currently available treatments for hearing loss, such as digital hearing aids and cochlear implants, do not promote regeneration of the auditory sensory epithelium (2). Because of a better understanding of the regenerative failure of the mammalian cochlea and re-epithelization of the non-mammalian auditory epithelium following injury, new approaches have been introduced for the treatment of hearing loss. One possible treatment to regenerate the auditory epithelium is transplantation of stem cells into the injured mammalian inner ear. According to the microenvironment theory, when organ-specific stem cells are transplanted into a damaged tissue, they might recognize intrinsic signals from the environment and consequently differentiate into cells of the host tissue (3).

In 2002, Malgrange et al. (4) observed the formation of cell colonies called spheres in suspension cultures of the dissociated auditory sensory epithelium of postnatal mice. These spheres had the property of self-renewal and expressed genetic markers of the developmental inner ear and nervous system. In addition, these spheres differentiated into various cell types including hair cells, supporting cells, and neurons. Using immunocytochemistry, these spheres were shown to be positive for stem cell markers nestin (4 5 6 7) and Sox2 (7,8). However, it remains unclear whether these progenitor/stem cells can serve as a source for cell replacement therapy of injured cochleas.

In this study, postnatal mouse inner ear progenitor/stem cells (mIESCs) were transplanted into the inner ears of neomycin-injured mature guinea pigs to investigate their viability. Our aim was to evaluate the potential role of mIESCs in hair cell replacement. We also examined the effects of mIESC transplantation on auditory functions by auditory brainstem response (ABR) measurement.

## Material and Methods

### Animals

Postnatal BALB/c mice were used as donors of mIESCs, and 8 female *Cavia porcellus* guinea pigs, weighing 250-350 g, were used as recipient animals. The experimental protocols were approved by the Internal Review Board on Ethics in Animal Research of the Faculdade de Medicina Universidade de São Paulo (1221/06 and 0466/08). Experimental procedures were performed in accordance with the National Institutes of Health Guidelines for the Care and Use of Laboratory Animals.

### Study overview

For the experimental procedures, we used eight guinea pigs with otoscopically healthy external and middle ears, and normal hearing status [40 dB sound pressure level (SPL), 16 kHz pure tone stimuli]. All animals were deafened by intratympanic delivery of a 10% neomycin solution at 7 days prior to mIESC transplantation. In neomycin-injured ears, a threshold increase of at least 30 dB SPL was confirmed by ABR immediately before stem cell transplantation. Prior to transplantation, mIESCs were transduced with a lentiviral vector carrying the *Lac-Z* reporter gene. At 2 weeks post-transplantation, ABR was measured to investigate the functional effects. The animals were sacrificed to remove the cochlea that was sectioned and subjected to histological analyses.

### Culture of mIESCs

For each experiment, temporal bones were dissected from eight mice. Each organ of Corti (OC) was dissected from the surrounding tissue (Reissner’s membrane, spiral ligament, *stria vascularis*, and remaining nerve fibers) and carefully rinsed in Hank’s balanced salt solution. OCs were individually treated with 0.25% trypsin/EDTA (Invitrogen, USA) in PBS at 37°C for 5 min. Enzymatic digestion was stopped by addition of trypsin inhibitor/DNAse I (Worthington Biochemical, USA). The tissue was dissociated by careful pipetting 30 times using filter tips (epTIPS Filter 20-300 µL; Eppendorf, Germany) and then diluted in 2 mL sphere culture medium (Dulbecco’s modified Eagle’s medium: F12 with N2, B27, basic fibroblast growth factor, insulin-like growth factor, epidermal growth factor, and heparin sulfate; Invitrogen). To confirm dissociation, cells were inspected under an Axiovert 40C inverted microscope (Carl Zeiss, Germany). The single cell suspension was passed through a 70-µm cell strainer (BD Biosciences, USA) to remove cell aggregates and debris. For sphere formation, the cell suspension was equally distributed into two wells of a six-well suspension culture plate (Greiner Bio-One, USA). The culture was maintained for 2 days at 37°C with 5% CO_2_ (7,9,10).

### Phenotypic characterization of mIESCs

After 2 days in suspension culture, spheres were transferred into eight-well Nunc Lab-Tek II chamber slides (Thermo Fisher Scientific, USA). To characterize undifferentiated spheres, they were incubated in the same culture medium overnight at 37°C with 5% CO_2_. Then, the spheres were fixed in 4% paraformaldehyde at room temperature (RT) for 20 min, followed by permeabilization in 0.3% Triton X-100/PBS for 20 min and incubation in blocking buffer [PBS containing 1% bovine serum albumin (BSA) and 10% goat serum; Santa Cruz Biotechnology, USA], for 1 h. The spheres were then incubated overnight at 4°C with mouse anti-nestin (Chemicon, USA) and goat anti-Sox2 (Chemicon) monoclonal antibodies diluted at 1:100 in blocking buffer. Secondary antibodies were Alexa Fluor 488-conjugated anti-mouse (1:500 dilution, Invitrogen) and Alexa Fluor 546-conjugated anti-goat (1:400 dilution, Invitrogen) antibodies. Two rinses in PBS were performed between each step. Samples were mounted in ProLong Gold antifade reagent (Invitrogen) containing 4′,6-diamidine-2-phenyl indol (DAPI) to visualize nuclei (7).

For differentiation, the spheres were transferred to chamber slides coated with poly-l-ornithine (0.1 mg/mL) and fibronectin (5 µg/mL) for adherent culture. The medium used for adherent culture was the same except for omission of the supplemented growth factors. Half of the medium was exchanged with fresh medium every 2 days. Cells were maintained in culture for 7 days to observe differentiated cells, followed by indirect immunocytochemistry. After fixation, permeabilization, and blocking, differentiated cells were incubated with primary antibodies against myosin VIIa (polyclonal rabbit, 1:100 dilution; Affinity BioReagents, USA), p27kip1 (monoclonal rabbit, 1:50 dilution; Abcam, UK) and βIII tubulin (monoclonal mouse, 1:250 dilution; Millipore, USA). Alexa Fluor 488-conjugated anti-goat (1:400 dilution, Invitrogen), Alexa Fluor 546-conjugated anti-rabbit (1:400 dilution, Invitrogen), and Cy3 anti-mouse (1:1000 dilution, Invitrogen) secondary antibodies were used to detect primary antibodies (7,9,10).

### Viral transduction of mIESCs

To identify transplanted cells in guinea pig cochleas, mIESCs were transduced with a lentiviral vector carrying the *lacZ* gene (Plasmid 12108 Mammalian Expression, Lentiviral; Addgene, USA). Construction of the *LacZ* lentivirus has been described previously (11). The *LacZ* reporter gene encodes β-galactosidase (β-gal) that catalyzes the reaction of the chromogenic substrate 5-bromo-4-chloro-3-indolyl-b-D-galactopyranoside (X-gal) to 5-bromo-chlorindoxyl that is subsequently converted to a blue product, 5-dibromo-4-dichloro indigo, in the presence of oxygen. In addition to the X-gal reaction, a monoclonal anti-β-gal antibody (Sigma Aldrich, USA) was used to detect β-gal expression in the transplanted tissue.

The spheres (DIV2) were centrifuged at 100 *g* for 5 min in a bench top centrifuge. The resulting cell pellet was re-suspended in a low volume of culture medium, the *LacZ* lentivirus was added at a multiplicity of infection of 5 in the presence of 8 µg/mL polybrene, and the cells were incubated for 3 h at 37°C and 5% CO_2_ (11). After transduction, 2 mL of medium were added to the spheres, followed by incubation for another 48 h. At the time of surgery, the transduced spheres were dissociated by enzymatic digestion and mechanical trituration. Then, 1×10^4^ cells were re-suspended in 10 µL aliquots of culture medium for transplantation.

One 10-µL aliquot was subjected to the X-gal-based cytochemical method to detect β-gal activity in the dissociated spheres and measure *LacZ* expression to determine the transfection rate. The dissociated spheres were transferred to chamber slides coated with fibronectin for adherent culture. The cells were centrifuged at 200 *g* for 5 min. After washing with PBS, the cells were incubated in 0.25% glutaraldehyde for 5 min at 4°C. After washing with PBS, the dissociated spheres were incubated at 37°C for 8 h in a reaction mixture containing 100 µg/mL X-gal (5-bromo-4-chloro-3-indolyl beta-d-galactopyranoside), 2 mM MgCl_2_, 20 mM potassium ferrocyanide (K_4_Fe(CN)_6_), 20 mM potassium ferricyanide (K_3_Fe(CN)_6_) (Sigma Aldrich), and 100 mM NaPO_4_ in PBS, pH 7.3 (11).

### Induction of hearing loss

After evaluation of normal hearing status (40 dB SPL, 16 kHz pure tone stimuli), all guinea pigs were deeply anesthetized with ketamine (40 mg/kg body weight, Ketalar^¯^) and xylazine (4 mg/kg body weight, Rompun^¯^), and then deafened by intratympanic injection of a 10% neomycin solution (prepared at the Divisão de Farmácia, Hospital das Clínicas, Faculdade de Medicina, USP) (12,13). Under an operating microscope (Zeiss OPMI¯ pico; Carl Zeiss), the tympanic membrane was exposed. The right middle ear was treated with 0.1 mL of the 10% neomycin solution via a 1-mL syringe attached to a 13×4.5 needle (12).

At 7 days after induction of deafness, animals that had auditory thresholds below 70 dB SPL were excluded from the study. Then, the deafened guinea pigs underwent transplantation microsurgery in the cochlea.

### Determination of the ABR threshold

To measure ABR thresholds, we used the Intelligent Hearing System and Smart-EP software (Intelligent Hearing Systems, USA), coupled to an HP-110 mini laptop. This software was designed to generate specific acoustic stimuli through high frequency transducers. After deep anesthesia, needle electrodes were inserted into the subcutaneous tissue of the vertex (active) and the ventrolateral regions of the right (reference) and left ears (ground). A probe was gently placed into the right external auditory canal. After checking the impedance (<3 kΩ), stimuli were delivered monaurally at a specific frequency of 16 kHz with calibrated ER2 Insert Earphones and High Frequency Transducers (Intelligent Hearing Systems). This specific frequency was chosen because it is the most affected by ototoxic insults (14).

The stimuli for each condition were presented at a rate of 19.1 times/s for a total of 1024 sweeps for each intensity. A gain of 100.000 was used with band pass-filtration below 100 Hz and above 3 kHz. The first tested stimulus intensity was 90 dB SPL. Stimulation levels were decreased in 10-dB steps initially and 5-dB steps around the hearing threshold that was determined by the lowest intensity at which all ABR waves were detectable. All intensities were checked twice to confirm the reproducibility of the response, and the thresholds were analyzed by two researchers (15,16) who were blinded to the experimental conditions.

After induction of hearing loss, participation of the left (control) ear was eliminated by introduction of a masking noise. Interaural attenuation in guinea pigs is 50 dB SPL at 16 kHz (17).

ABR recordings were carried out at three time points: immediately before induction of deafness to confirm normal hearing, at 7 days after induction of deafness and immediately before mIESC transplantation into the cochlea to confirm an increase in hearing thresholds, and at 2 weeks after mIESC transplantation into the cochlea to evaluate post-operative hearing.

### Microsurgery for mIESC transplantation into the cochlea

All surgical procedures were performed under aseptic conditions. The microsurgical techniques were performed under an operating microscope (Zeiss OPMI¯ pico). To reduce the risk of postoperative infection, the transplanted animals received an intramuscular injection of potassium benzylpenicillin (100,000 U/kg body weight) before surgery. In addition to the procedures previously described for induction of deep anesthesia, a local anesthetic (2% lidocaine) was subcutaneously delivered to the incision site. All transplantations were performed in the right cochlea. The left cochlea served as a control. A retroauricular incision was made and the otic bulla was exposed (18). The bony wall of the bulla was partially drilled to expose the basal turn of each cochlea. A small hole was then made in the lateral wall at the basal turn of the cochlea, corresponding to the location of the scala tympani. To avoid additional cochlear trauma, 5 µL perilymph was removed slowly. Then, 10 µL culture medium containing *LacZ*-lentivirus-transduced mIESCs (1×10^4^ cells/µL) or culture medium only were infused into the scala tympani using a microsyringe (26 G Hamilton syringe) at a speed of 5 µL/min. The cochleostomy was sealed with a muscle plug, and the skin wound was closed with sutures (12,19,20).

The animals were divided into two experimental groups each containing four animals: group A (study group) consisted of neomycin-injured animals transplanted with culture medium containing *LacZ-lentivirus*-transduced mIESCs, and group B (control group) consisted of neomycin-injured animals transplanted with culture medium only. To reduce systematic bias in the transplantation procedure, the same surgeon operated on guinea pigs in both groups. The surgeon was blinded to the contents of the injections.

### Immunohistochemistry

Two weeks after mIESC transplantation, guinea pigs from the study and control groups were euthanized by an overdose of ketamine and xylazine via intramuscular injection, followed by CO_2_ inhalation. Whole cochleas were removed and fixed in 4% paraformaldehyde. The cochleas were then decalcified in 10% formic acid for 24 h at RT. To obtain consecutive frozen sections of 16 µm (parallel to the longest axis, CM-1850; Leica), fixed tissue was incubated in 30% sucrose overnight at 4°C, placed in Tissue Tek O.C.T. compound (Sakura Finetek, The Netherlands), and cooled to -80°C. The sections were permeabilized by incubation in 0.2% Triton X-100 for 30 min at 4°C and then exposed to a blocking solution consisting of 5% horse serum and 5% BSA in PBS for 2 h at 4°C. After two washes in PBS, the samples were incubated overnight at 4°C with primary antibodies in 100 mM lysine and 0.2% Triton X-100 in PBS. After several washes in PBS, the specimens were incubated with fluorescent dye-conjugated secondary antibodies for 2 h at RT. Primary antibodies were polyclonal anti-myosin VIIa antibody (Affinity BioReagents) and monoclonal anti-β-gal antibody (Sigma Aldrich) both diluted at 1:100. Secondary antibodies were Alexa Fluor 488-conjugated goat anti-rabbit (Invitrogen) and AlexaFluor 568-conjugated goat anti-mouse (Invitrogen) both diluted at 1:200. The sections were coverslipped using ProLong Gold antifade reagent (Invitrogen) containing DAPI for nuclear staining. Images were obtained under a fluorescence microscope (Axioplan, Carl Zeiss) or confocal microscope (LSM 510, Carl Zeiss). Some sections were also stained with hematoxylin and eosin (HE) using standard procedures, and images were acquired under an optical microscope (Axioplan) (21,22).

### Cell fate analyses of transplanted mIESCs

Expression of the *LacZ* reporter gene was assessed by a colorimetric assay to detect β-gal activity or immunofluorescence to detect LacZ as described above. Because the structure of the basal turns was intact in all sectioned cochleas, the number of transplanted cells was counted in four anatomical subregions of the scala media: lateral wall, basilar membrane, limbus spiral, and the endolymph. The distribution of transplanted cells was compared in each compartment. We counted the number of DAPI-stained cells expressing β-gal as transplanted cells. Transplanted cells co-expressing myosin VIIa and β-gal were considered as “hair cell like” (22).

Four longitudinal mid-modiolar sections (each separated by 16 µm) were counted in each animal. To avoid counting the same cells more than once, only non-adjacent serial sections were used for cell counting. The sections were viewed under an epi-fluorescence microscope (Axioplan) or confocal microscope (LSM 510). Contralateral control cochleas were also examined.

### Statistical analysis

Data are reported as means±SD. Significant differences in mean threshold values were determined using the Wilcoxon signed-rank test for comparison of ABR thresholds before and after neomycin delivery, and comparison of ABR thresholds in control and study groups. P<0.05 was considered to be statistically significant. Statistical analyses were performed using SPSS Statistics, Version 20 (IBM, USA).

## Results

### Postnatal mouse inner ear-derived cells have stem cell properties

**Figure 1:**
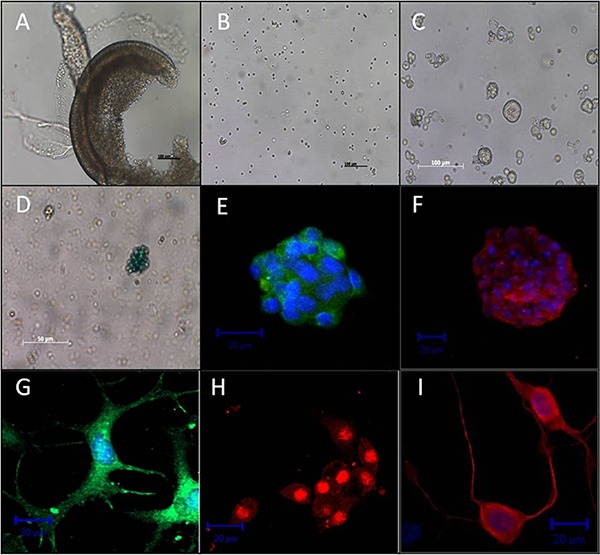
Mouse inner ear progenitor/stem cells (mIESCs) express stem cell markers and differentiate into cells expressing hair and supporting cell markers under certain conditions. A cochlear duct dissected from the modiolus, containing (*A*) the organ of Corti, spiral ligament/stria vascularis, and Reissner’s membrane. Dissociation of the organ of Corti yielded a suspension of cells as observed in culture (*B*). After 2 days in suspension culture, floating colonies of cells (*C*) were round and compact (solid spheres). *LacZ*-lentivirus-transduced mIESCs were discerned by blue staining (*D*) in X-gal assays. Under suspension culture conditions (*E-F*), spheres contained cells expressing Sox2 (*E*, green) or nestin (*F*, red), markers of pluripotency and neural stem cells, respectively. Upon differentiation under adherent conditions (*G-I)*, sphere cells expressed myosin VIIa (*G*, green) and p27kip1 (*H*, red), markers of hair and supporting cells, respectively. Cells with a neuronal phenotype, indicated by βIII-tubulin staining, were also observed (*I*, red). Nuclear DNA was stained with 4′,6-diamidine-2-phenyl indol (blue) (*E*, *F*, *G*, *I*). Scale bars: 100 µm (*A-C*); 50 µm (*D*); 20 µm (*E-I*).

As previously demonstrated by our group (7), after 2 days in suspension culture, isolated cells (Figure 1B) from the postnatal mouse OC (Figure 1A) give rise to floating clonal colonies (spheres) capable of propagating into additional spheres upon serial passaging (n=2 passages, Figure 1C). These spheres were round, compact, and contained densely packed small cells in their interior. Therefore, they are called solid spheres (9).

Using indirect immunofluorescence, we immunolabeled Sox2 (Figure 1E) and nestin (Figure 1F) in the solid spheres. Nestin is an intermediate filament protein expressed in neuroepithelial stem cells (23), and Sox2 is a universal marker of stem cells (24).

To determine whether sphere cells could differentiate into hair and supporting cells, we induced differentiation by omission of growth factors in adherent culture conditions for 7 days. Consistent with recent findings (4,7,9,25), the cells expressed myosin VIIa (Figure 1G) and p27kip1 (Figure 1H), markers of hair and supporting cells, respectively. Cells with large nuclei and long, thin processes suggestive of a neuronal phenotype were identified by immunostaining of βIII-tubulin, a mature neuron marker (Figure 1I). Taken together, these results are consistent with previous studies showing that postnatal mouse inner ear-derived sphere cells have the properties of stem cells.

### Efficiency of viral transduction *in vitro*

Lentiviral vectors mediate efficient gene delivery *in vitro* and *in vivo*, undergo genome integration, and promote long-term expression of transgenes in non-dividing cells (26). In our study, after *lacZ-*lentivirus transduction, sphere cells were positive for *LacZ* activity as determined by a colorimetric assay (Figure 1D). Overall, 85% of cells were positive for LacZ (data not shown).

### Efficiency of hearing loss induction by neomycin in guinea pigs

**Figure 2:**
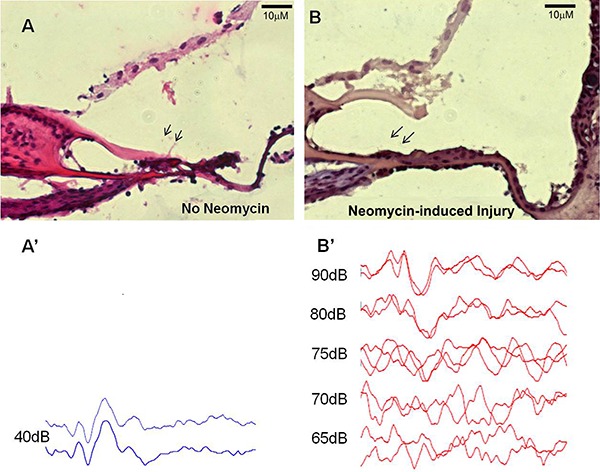
Neomycin treatment damages the cytoarchitecture of the organ of Corti and causes auditory dysfunction. Longitudinal sections of guinea pig cochleas subjected to transtympanic neomycin treatment had a basilar membrane with a flat epithelium (*B*) compared with the control (*A*) in which the tectorial membrane directly joined to the whole organ of Corti (H&E). *A* and *B* are mirror images. At 7 days after neomycin treatment, significant increases in auditory brainstem response threshold levels for pure 16-kHz stimuli were registered for the right ears of treated animals (*B′*) compared with the control group (*A′*).

The most sensitive frequency to ototoxic injury is 16 kHz (27). The hearing status of all guinea pigs was confirmed to be normal by measuring the ABR to 40 dB SPL pure tone (16 kHz) stimuli (Figure 2). All guinea pig right ears were deafened by an injection of 10% neomycin through the tympanic membrane (12). To assess the extent of hearing loss, ABR threshold levels were tested at 7 days after neomycin treatment and compared with pre-treatment levels. Significant auditory threshold shifts of 35.6 ± 4.2 dB SPL were observed in treated animals exposed to pure tone (16 kHz) stimuli (P<0.05; Table 1).

**Table:**
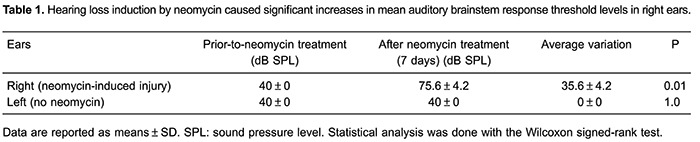

Histologically, the OC and other specialized structures of the cochlear duct are usually severely injured after treatment with neomycin (14,28). In our study, we did not observe the complex cellular organization of the OC, including the inner and outer hair cell rows and specialized supporting cells. The epithelium on the basilar membrane became simple, and the cells were cuboidal or flat. Another anatomical change was detachment of the tectorial membrane from outer hair cell stereocilia bundles (Figure 2).

### Control and study groups exhibited similar auditory thresholds before and after transplantation

To evaluate the effect of mIESC transplantation on the auditory thresholds of deafened guinea pigs, we assessed ABRs at two time points: before surgery and at 2 weeks after surgery. Prior to surgery, the auditory thresholds of animals in both groups were similar. For pure tone (16 kHz) stimuli, the ABR threshold shift between pre- and post-surgery levels did not differ significantly between the two groups (P>0.05; Table 2).

**Table:**
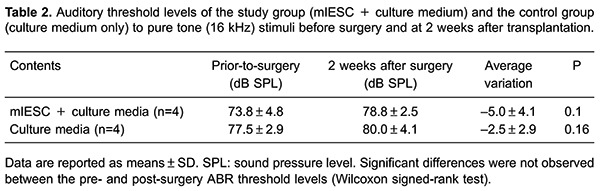

### Transplanted mIESCs survived and integrated into the cochlea

In the study group, mIESCs were transplanted into the right cochlea of four guinea pigs at 7 days after neomycin injury. Animals were sacrificed at 2 weeks after transplantation. The right cochlea of one animal had purulent secretions in the middle ear cavity. Therefore, this animal was excluded from the study. Basal turns of the three remaining cochleas were analyzed. Although not a primary objective of the study, there was no sign of pronounced infiltration of inflammatory cells into the transplanted cochleas.

**Figure 3:**
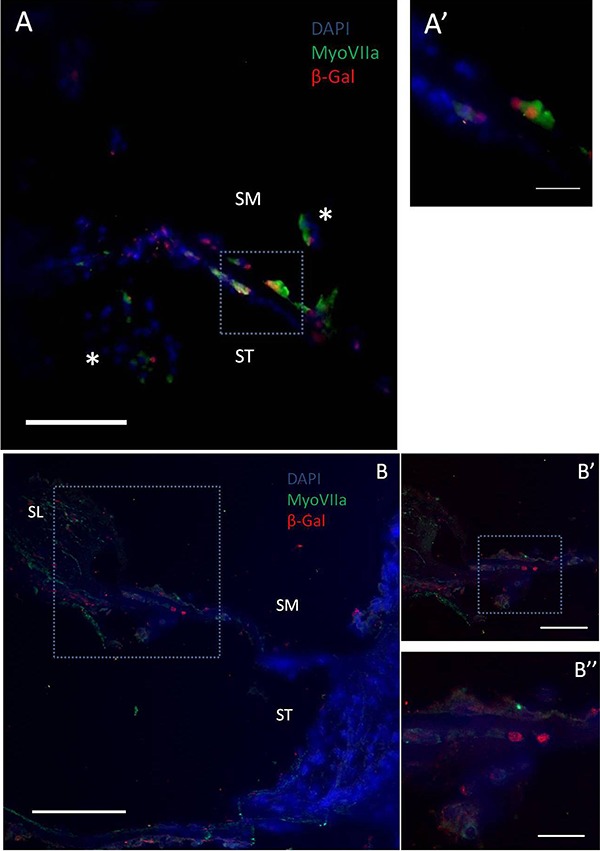
. Some mIESCs integrated into the neomycin-injured cochlea and expressed a hair cell marker. Transplanted mIESCs were detected in the neomycin-injured cochleas of guinea pigs as shown by indirect immunofluorescence of β-galactosidase (β-gal) (red) to detect the protein expressed by the bacterial *LacZ* reporter gene. Myosin VIIa (hair cell marker) was detected by a specific antibody (green). Nuclear DNA was stained with DAPI (blue). Some transplanted mIESCs (β-gal+) were positive for myosin VIIa. Most transplanted mIESCs were found in clusters (asterisks in *A*) in the endolymphatic space, and a small number of these cells were integrated into the basilar membrane (*A′* and *B-*). *A* and *B* are images of cochleas from two animals. Higher magnification views of the regions defined by the square in *A* and *B* are shown in *A′* and *B′-B-*, respectively. ST: scala tympani; SM: scala media; SL: spiral limbus. Scale bars: 100 µm (*B*); 50 µm (*A* and *B′*); 20 µm (*A′* and *B-*).

**Figure 4:**
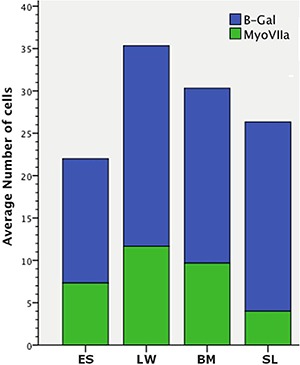
Average number of transplanted mIESsC expressing β-gal and the number of cells expressing β-gal + myosin VIIa in various areas of the scala media. ES: endolymphatic space; LW: lateral wall; BM: basilar membrane; SL: spiral limbus.

After sacrifice, histological analysis of the cochlea was performed to identify LacZ-positive cells, which would indicate the survival and integration of mIESCs into the host cochlea. The localization of the cells was examined in the scala tympani and elsewhere in the cochlea. Cell clusters (spheres) containing transplanted LacZ-positive mIESCs were found in all scalae of basal turns: media, tympani, and vestibuli. In the scala media, the target site where the OC lies, transplanted mIESCs were found in the lateral wall, basilar membrane, spiral limbus, and endolymphatic space (Figure 3). No significant differences were found in quantitative analysis of the transplanted mIESCs among regions of the scala media (P>0.05; Figure 4). No mIESCs were detected in the basal turns of contralateral control cochleas.

Of the transplanted mIESCs in the scala media, 42.6±5.7% were positive for the hair cell marker myosin VIIa. There were no significant differences in the ratio of transplanted mIESCs expressing myosin VIIa among the regions of the scala media: lateral wall, basilar membrane, spiral limbus, and endolymphatic space (P>0.05; Figure 4).

## Discussion

Stem cell transplantation is a promising approach for hearing loss therapy. In recent years, several types of stem cells have been used in related studies. Neural (22), bone marrow (29), and embryonic (30,31) stem cells are the most widely used for transplantation. These studies aimed to replace lost hair cells or spiral ganglion neurons. The transplanted cells were expected to exhibit the desired phenotypes, integrate, and establish adequate functional connections within the host cochlea (32).

The choice of stem cell type for transplantation plays a crucial role in the outcome. In our study, we used inner ear stem cells derived from the postnatal mouse OC, which express stem cell markers (nestin and Sox2) and are able to differentiate into hair and supporting cells *in vitro* (Figure 1). Because of their cochlear origin, mIESCs are probably the cell type that most accurately recapitulates normal differentiation processes in the OC *in vivo* (2). Thus, mIESCs may be a valuable cell source to examine the viability of differentiated hair cells in the injured inner ears of animal models. In addition, our study is the first to follow the fate of transplanted mIESCs in cochleas damaged by ototoxic treatment.

Several ototoxic treatments lead to the loss of hair cells, thus increasing auditory thresholds. Application of neomycin to the tympanic cavity induces the death of most hair cells and provides an excellent model of induced hearing loss (12). In this study, at 1 week after neomycin application, there was remarkable injury to hair cells (Figure 2B) and an increase in ABR thresholds (Figure 2B′). It is hypothesized that the damaged tissue releases factors that enhance the survival and differentiation of transplanted cells in an attempt to stimulate endogenous repair mechanisms (33).

Immunological rejection of transplanted cells could have occurred in our experiments because we used a xenotransplantation procedure and none of the animals received immunosuppressants. We performed xenotransplantation because, in contrast to the mouse inner ear, the postnatal guinea pig inner ear has a limited capacity to give rise to spheres with stem cell properties such as expression of stem cell markers, differentiation, and self-renewal (7). An adult guinea pig model was chosen because it is more reliable for ototoxicity-induced hearing loss than a mouse model (14). Although we expected immunological rejection, there was no evidence of infiltration by inflammatory cells at 2 weeks after transplantation. Similar studies have used cross-species transplantation with no reported adverse immune reactions, leading to claims that the cochlea displays immunological privilege that allows xenotransplantation (33,34). Another interesting aspect is the fact that the cochleostomy did not affect the viability of the transplanted cells.

Our findings demonstrated that mIESCs have the potential to migrate into the injured cochlea because they were found in other scalae (vestibuli and media) in addition to the scala tympani. Migration from the scala tympani to vestibuli could be attributable to the flow of perilymph (33). To reach the scala media, transplanted cells migrated through transiently damaged areas of the basilar membrane (Figure 2) (2).

Despite the potassium-rich endolymph of the scala media, some transplanted mIESCs were detected in the basilar membrane, and some of these cells expressed myosin VIIa. These integrated mIESCs expressing a hair cell marker were not able to replace the complex cytoarchitecture of the OC (Figure 3). This finding is in accordance with the previously published hypothesis regarding a sheet of hair cells repopulating the damaged OC epithelium, which is similar to the organization of the avian basilar papilla. The theory suggests that these generic hair cells can provide trophic factors for spiral ganglion neurons and enhance the performance of current cochlear implants (35).

In our study, some mIESCs localized in the lateral wall and spiral limbus expressed myosin VIIa, while endogenous cells of these regions failed to express this marker. Similar findings were obtained by transplantation of neural stem cells into the scala tympani (22). This unusual expression of myosin VIIa could be attributable to environmental cues in the transplanted cochlea and/or initial cellular differentiation prior to surgery during viral transduction *in vitro*.

Despite cellular integration into the basilar membrane, functional evaluation of mIESC-transplanted guinea pigs revealed no significant improvement in auditory thresholds. This finding could be attributable to the short observation period (2 weeks), during which the formation of functional synaptic contact with new cells showing a hair-cell phenotype was unlikely. Similarly, previous studies of various experimental models have reported no functional gain of auditory functions after stem cell transplantation.

Various approaches are necessary to determine the steps needed for hair cell repair in the OC and restoration of auditory functions. Although considerable advances have been made toward elucidating the genetic determinants of the mIESC cycle and differentiation, there is still a lack of information on aspects that will contribute to homing, long-term *in vivo* viability of transplanted mIESCs engrafted in the OC, as well as differentiation into hair cells.

In our experimental model, we confirmed transtympanic administration of neomycin as an effective method to induce hearing loss in the guinea pig cochlea, and the lentiviral *LacZ* gene as a useful reporter to follow the fate of mIESCs. At 2 weeks after surgery, no significant effect was observed on auditory functions of cochleas that received mIESCs. However, we present evidence for the viability and engraftment of mIESCs in the OC of injured cochleas.
